# Predicting ICU Readmission from Electronic Health Records via BERTopic with Long Short Term Memory Network Approach

**DOI:** 10.3390/jcm13185503

**Published:** 2024-09-18

**Authors:** Chih-Chou Chiu, Chung-Min Wu, Te-Nien Chien, Ling-Jing Kao, Chengcheng Li

**Affiliations:** 1 Department of Business Management, National Taipei University of Technology, Taipei 106, Taiwan; chih3c@ntut.edu.tw (C.-C.C.); cmwu@ntut.edu.tw (C.-M.W.); lingjingkao@ntut.edu.tw (L.-J.K.); 2 College of Management, National Taipei University of Technology, Taipei 106, Taiwan; chengchengli0006@gmail.com

**Keywords:** ICU readmission, electronic health records, BERTopic, LSTM network, deep learning, healthcare

## Abstract

**Background:** The increasing rate of intensive care unit (ICU) readmissions poses significant challenges in healthcare, impacting both costs and patient outcomes. Predicting patient readmission after discharge is crucial for improving medical quality and reducing expenses. Traditional analyses of electronic health record (EHR) data have primarily focused on numerical data, often neglecting valuable text data. **Methods:** This study employs a hybrid model combining BERTopic and Long Short-Term Memory (LSTM) networks to predict ICU readmissions. Leveraging the MIMIC-III database, we utilize both quantitative and text data to enhance predictive capabilities. Our approach integrates the strengths of unsupervised topic modeling with supervised deep learning, extracting potential topics from patient records and transforming discharge summaries into topic vectors for more interpretable and personalized predictions. **Results:** Utilizing a comprehensive dataset of 36,232 ICU patient records, our model achieved an AUROC score of 0.80, thereby surpassing the performance of traditional machine learning models. The implementation of BERTopic facilitated effective utilization of unstructured data, generating themes that effectively guide the selection of relevant predictive factors for patient readmission prognosis. This significantly enhanced the model’s interpretative accuracy and predictive capability. Additionally, the integration of importance ranking methods into our machine learning framework allowed for an in-depth analysis of the significance of various variables. This approach provided crucial insights into how different input variables interact and impact predictions of patient readmission across various clinical contexts. **Conclusions:** The practical application of BERTopic technology in our hybrid model contributes to more efficient patient management and serves as a valuable tool for developing tailored treatment strategies and resource optimization. This study highlights the significance of integrating unstructured text data with traditional quantitative data to develop more accurate and interpretable predictive models in healthcare, emphasizing the importance of individualized care and cost-effective healthcare paradigms.

## 1. Introduction

The COVID-19 pandemic has created significant challenges for global medical units, particularly intensive care units (ICUs), due to the increasing number of infected patients. Hospitals must allocate resources, such as equipment and labor, wisely and make informed decisions based on the available information and resources in the ICU. The World Health Organization recommends that hospitals regularly monitor specific clinical variables of COVID-19 patients and analyze them using medical technology to aid in decision-making [1]. However, since patients’ illnesses change rapidly, clinicians face the challenge of making quick and accurate decisions without adequate and up-to-date information [2]. Patients in the ICU are often in critical condition and are at a higher risk of death than patients in other hospital departments. Additionally, readmissions and prolonged hospital stays are common clinical outcomes that can indicate the patient’s health status, critical care quality, and medical efficiency. Readmission soon after discharge threatens the quality of patient care and increases healthcare costs.

Nevertheless, Electronic Health Records (EHRs) have become a foundational resource for broader analysis and prediction, encompassing medical records, electrocardiograms, and medical imaging, providing researchers with a rich data source to support critical clinical decision-making [3]. The electronic archiving of EHR data has not only improved hospital management and service delivery but also provided valuable resources for related research, such as predictive modeling [4,5,6]. In the critical care setting, several widely used scoring systems have been developed to assess the severity of a patient’s condition and predict in-hospital mortality risk. These scoring systems, such as the Sequential Organ Failure Assessment (SOFA) [7], Acute Physiology and Chronic Health Evaluation (APACHE) [8,9], Mortality Probability Model (MPM) [10,11], and Simplified Acute Physiology Score (SAPS) [12,13,14], are invaluable in clinical practice. They assist healthcare professionals in making informed decisions, optimizing treatment strategies, and providing quantitative measures for monitoring patient conditions. However, while many studies have utilized EHR data, the majority have focused solely on quantitative EHR data [15,16,17,18,19]. In reality, approximately 80% of EHR data is in text form, including free-text notes and clinician progress notes, which document patients’ physiological states during their visits [20].

In recent times, there has been a profusion of groundbreaking advancements in the exploration of textual semantics using natural language processing models. Among these, Latent Dirichlet Allocation (LDA) has garnered significant attention. Moreover, Ozmen et al. [21] underscore the efficacy of LDA in discerning perilous occurrences via natural language processing models. Conversely, Vaughn et al. [22] employ LDA to predict hospitalization and biological outcomes among inflammatory bowel disease (IBD) cohorts, resulting in substantial improvements. However, the LDA framework imposes certain limitations, notably its incapacity to consider the nuanced arrangement of words within a document due to its inherent unigram text model and the absence of independence and orthogonality among the generated topics stemming from the recurrence of identical words across multiple topics [23,24].

To transcend these constraints and fully harness the potential of EHR data, this investigation advocates for the embrace of BERTopic, an innovative topic modeling technique that harnesses BERT (Bidirectional Encoder Representations from Transformers) embeddings and c-TF-IDF to construct dense clusters and expound upon easily interpretable topics [25]. Unlike LDA, BERTopic enables a semantic representation of each term, thereby alleviating vocabulary mismatch issues and illuminating changes in distribution by topic over time. BERTopic necessitates less hyperparameter tuning than LDA and integrates noise topics to prevent the misassignment of irrelevant documents to other topics, thus enhancing topic representations. Recent scholarly endeavors, such as those of Uncovska et al. [26], have spearheaded the utilization of BERTopic for sentiment analysis and topic modeling within the mHealth research domain. Similarly, Jeon et al. [27] applied BERTopic and PatentSBERT to patents registered with the United States Patent and Trademark Office, assessing the potential technological prowess based on thematic attributes concerning digital capabilities and resemblance to Digital Therapeutics (DTx) technologies. Wang et al. [28] employed BERTopic to identify interdisciplinary themes from extensive academic literature and subsequently conducted fine-grained evolution analysis on these extracted themes. The collective body of research underscores the promising applications of BERTopic.

To demonstrate the effectiveness of this approach, our study utilized comprehensive clinical data from the MIMIC-III repository on patients admitted to the ICU at Beth Israel Deaconess Medical Center in Boston, MA. The dataset contains detailed patient data, including laboratory test data, demographic characteristics, microbiology test results, treatment course, and fluid volumes in and out. The MIMIC-III dataset has been widely employed in numerous studies and publications spanning diverse domains, including clinical prediction, disease diagnosis, and treatment. Beyond the conventional utilization of structured data, existing literature highlights the effectiveness of integrating text, visual, and verbal signals to enhance patient clinical prediction [29,30,31,32]. Our study leverages extensive clinical data from the MIMIC-III dataset repository, specifically targeting quantitative and textual messages from ICU patients in the 24 h before discharge, with the goal of predicting patient readmission within 30 days of discharge. We introduce an innovative approach for ICU readmission prediction using a hybrid BERTopic and Long Short-Term Memory (LSTM) network, called BERTopic-LSTM. This study highlights the need for BERTopic and LSTM methods in healthcare predictive models, which offer promising avenues for improving patient care and outcomes. 

To ensure the transparency and rigor of our research, this study adheres to the STROBE (Strengthening the Reporting of Observational Studies in Epidemiology) guidelines [33]. STROBE provides a structured framework for reporting observational studies, emphasizing clarity in the presentation of research objectives, methods, and findings [34]. By following these guidelines, our study aims to improve the reproducibility and comparability of our results with other studies in the field. Employing STROBE helps us to systematically detail the data collection and analysis processes, facilitating a clear understanding for other researchers and practitioners in the field. The adherence to these guidelines underlines our commitment to maintaining the highest standards of research integrity and methodological rigor, which are particularly crucial in studies involving complex datasets like MIMIC-III. 

Our research introduces an innovative approach for predicting ICU readmissions by integrating both quantitative and text data to enhance predictive capabilities. Our approach combines BERTopic with LSTM networks, significantly improving the interpretability and accuracy of the model. By transforming discharge summaries into topic vectors, our model enhances prediction transparency and supports personalized care. The topics generated through BERTopic effectively guide the selection of key predictive factors, further enriching the model’s explanatory power. Additionally, the use of advanced analytical techniques, such as importance ranking, provides crucial insights into the interactions between input variables and readmission predictions, contributing to more efficient patient management and optimized resource allocation. This model empowers healthcare providers to implement targeted interventions, optimize follow-up care, and make data-driven decisions that improve patient outcomes. By incorporating these insights into routine clinical practice, medical institutions can better manage patient care, deliver higher quality services, and ultimately enhance healthcare quality while reducing costs.

## 2. Materials and Methods

### 2.1. Proposed Framework

**Figure 1:**
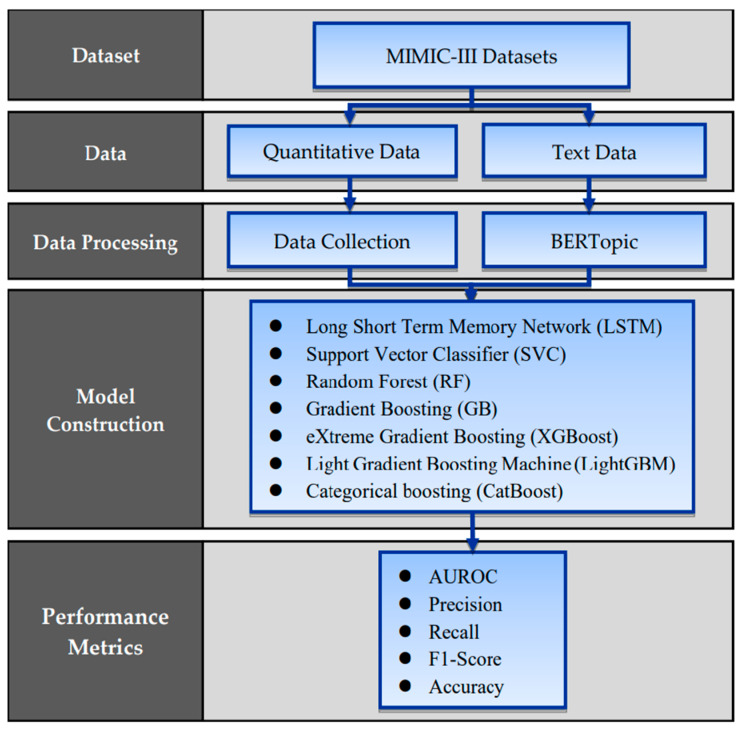
The detailed process of data extraction.

This study collected both quantitative and text data from hospitalized patients. Quantitative data primarily consisted of vital sign data observed within 24 h prior to ICU discharge and basic patient information collected during hospitalization. The collected data underwent data filtering and preprocessing. Text data were derived from patient discharge summaries and analyzed using BERTopic technology to generate themes. To model readmissions of ICU patients within 30 days of discharge, this study employed six commonly used machine learning methods, integrating quantitative and text data predictions. Finally, the models’ predictive performance was evaluated using five different metrics. The research framework is illustrated in Figure 1.

### 2.2. Data Collection and Preprocessing 

The Medical Intensive Care Information Mart (MIMIC-III) database contains comprehensive clinical data on patients admitted to Beth Israel Deaconess Medical Center in Boston, Massachusetts, and has become a major source of anonymized public data for research [35]. MIMIC-III contains 26 tables in CSV format, which record the data of patients during ICU treatment in detail, including laboratory examination data, demographic characteristics, microbiological examination results, circulation during hospitalization, treatment process, fluid volume in and out, etc. The large amount of clinical data contained in MIMIC-III makes it an invaluable resource for research in the medical field. In this study, we analyzed 46,520 patient-related data items and 58,976 admission-related data items such as vital signs, medications, laboratory measurements, and observation records using MIMIC-III. The average age of the adults in this dataset was 65.8 years, the length of ICU stay was 2.1 days, the length of hospital stay was 6.9 days, and the mortality rate during admission was 11.5%. We obtained ethical approval to access the MIMIC-III database by completing the National Institutes of Health (NIH) online course, passing the Human Research Participant Protection Exam, and submitting a request for access (certificate number: 35628530), thereby ensuring that appropriate safeguards were in place for all data used in this study.

To ensure that our results were comparable to those of previous related studies, we followed the same principles of patient selection and did not analyze the specific disease of the patients. Instead, we used data from all patients in our analysis, which increased the generalizability of our findings. This approach allowed us to contribute to the existing literature on predicting ICU readmission rates while maintaining consistency with previous studies [36,37,38]. The aim of this study was to predict ICU readmission within 30 days of ICU discharge. First, we excluded patients who were younger than 16 years of age at discharge (N = 8119), second, patients who had an ICU stay of less than 24 h (N = 2030), and finally, patients who died during hospitalization (N = 4846) were also excluded. Therefore, we obtained data on 43,981 patients who met the screening criteria.

The study utilized vital sign data obtained during the 24 h prior to patient discharge, which included Heart Rate (X_1_), Respiratory Rate (X_2_), Diastolic Blood Pressure (X_3_), Systolic Blood Pressure (X_4_), Temperature (X_5_), Oxygen Saturation (X_6_), Blood Urea Nitrogen (X_7_), Creatinine (X_8_), Non-Invasive Blood Pressure mean (X_9_), Glucose (X_10_), White Blood Cell count (X_11_), and GCS Eye (X_12_), GCS Motor (X_13_), and GCS Verbal (X_14_). Additionally, basic demographic variables such as Age (X_15_), ICU Length of Stay (X_16_), Hospital Length of Stay (X_17_), Gender (X_18_), Admission Type (X_19_), Discharge Location (X_20_), Insurance Type (X_21_), and Last ICU Type (X_22_) were considered. In total, 22 structural predictors were used in this study. Appendix A shows the selected variables.

In our study focusing on predicting mortality in ICU patients, managing missing data was a critical step due to the complexity of the datasets involved. We implemented a rigorous three-stage process for managing missing data, as referenced in our methodology [39]. The initial stage of data preprocessing involved a comprehensive assessment of the dataset’s completeness, where patient records exhibiting more than 30% missing values were identified and removed, totaling the exclusion of 7172 records. This procedure ensured that the remaining dataset was both more complete and reliable for subsequent analyses. In the second stage, we scrutinized the predictors used in our analysis. Predictors with missing values exceeding 40% were deemed unreliable for generating robust outcomes and were consequently removed to minimize potential biases from poorly populated data fields. The third and final stage entailed a meticulous cleanup of the statistical data, discarding any data that still exhibited more than 20% missing values according to the criteria set in the earlier stages, thus maintaining high data quality across all variables in the model. For the remaining missing values, a mean imputation technique was employed by calculating the mean of available values for each variable and substituting these means for the missing entries. This method was chosen because it preserves the overall distribution of the dataset and offers a straightforward and statistically sound approach to handling missing data.

**Figure 2:**
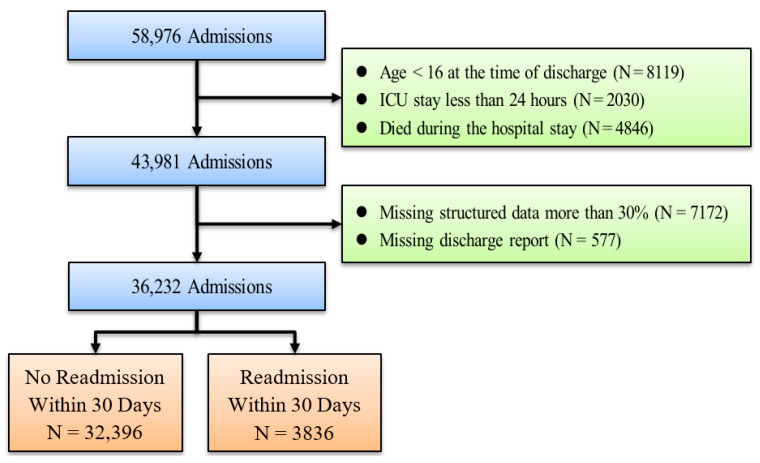
The process of data extraction.

Regarding text data management and ICU readmission criteria, our approach utilized discharge summaries created by attending physicians to supplement our data pool. However, a subset of patients lacked current discharge summaries, specifically 577 individuals. We employed specific selection criteria to identify ICU readmissions, including those readmitted or deceased within 30 days post-discharge, based on references [32,33]. Our analysis encompassed a total of 36,232 admissions, with 3836 ICU patients readmitted within this 30-day window, resulting in a readmission rate of 10.59%. This detailed methodology not only underscores the rigorous data management practices implemented but also highlights the careful consideration given to the unique aspects of ICU patient data, ensuring robust and reliable predictive modeling in our study. Figure 2 illustrates the data extraction process employed in our study.

Imbalanced datasets pose significant challenges for conventional learning algorithms and are prevalent in many application domains. When the class distribution is highly skewed, machine learning problems become unbalanced. In such cases, the results of a model can be negatively affected, reducing its predictive ability [40]. To address these issues, several solutions have been proposed. The Synthetic Minority Oversampling Technique (SMOTE) is a potent classification imbalance solution that generates new minority class samples by randomly sampling from the nearest neighbor line connecting the minority class samples [41]. SMOTE is extensively used to process skewed data and creates a balanced dataset by adding synthetic data to the minority class. Addressing imbalanced datasets is critical for effective machine learning. SMOTE is a powerful technique for generating synthetic data to balance class distributions, and under- and oversampling larger and smaller samples can improve predictive performance [42]. In our study, we employed SMOTE methodologies with varying proportions to address the issue of class imbalance. We conducted a series of tests using different ratio settings between readmission and non-readmission patients, including 1:1, 1:5, and 1:10. The results demonstrated that the model performed optimally under the 1:1 ratio setting, which was subsequently adopted for our predictive analysis. Specifically, by applying SMOTE with an augmentation factor of 800%, we significantly increased the number of instances in the “Readmission within 30 Days” class from 3836 to 30,688. This adjustment effectively enhanced the representation of the minority class, raising its proportion from 10.59% in the original dataset to 48.65% in the SMOTE-enhanced dataset.

### 2.3. BERTopic

BERTopic is a novel topic-modeling algorithm that employs a pre-trained language model, BERT, to extract topics from textual data [43]. This algorithm has demonstrated promising results in a variety of natural language processing tasks [44]. The BERT embedding maps a text sequence to a high-dimensional vector representation using a pre-trained BERT model. The process involves tokenizing and encoding the text, computing a sequence of context-dependent vector representations for each token using multiple layers of bidirectional transformer encoders, and averaging these vector representations to obtain a vector representation for the entire text sequence as the BERT embedding result. Mathematically, the BERT embedding for a text sequence is obtained by averaging the sequence of hidden states, where *h_i_* is the context-dependent vector representation for the *i*-th token:

(1) $$BERT\left(X\right)=\left(\frac{1}{n}\right)\;*\;\sum h_{i}$$

To better capture the key information in short texts and improve the performance of text classification and clustering, an improved version of TF-IDF, c-TF-IDF, has been developed. c-TF-IDF determines the importance of a word by calculating its inverse document frequency of classes (IDF-C). Specifically, IDF-C is the logarithmic inverse ratio of the number of documents containing at least one vocabulary in a category to the total number of documents. Moreover, c-TF-IDF uses log-normalized term frequency (TF) to reduce the impact of highly frequent words on the weighting. BERTopic leverages BERT and c-TF-IDF to generate interpretable topics [25]. The TF-IDF is given by Equation (2). Where the weight of the word *i* in the text (document) *j* is *W_ij_*. A is the average number of a word per class, *f_i_* is the frequency of word *i* across all classes, and *tf_ij_* is the frequency of the word *i* in the document *j*.

(2) $$W_{ij}=tf_{ij}\times log\left(1+\frac{A}{f_{i}}\right)$$

The BERTopic algorithm comprises three primary stages to generate a distribution of topics across a set of documents. The first stage involves constructing a similarity matrix among the documents using a pre-trained transformer model. The second stage involves reducing the dimensionality of the similarity matrix using UMAP. The third stage involves clustering the documents using HDBSCAN, and the resulting clusters are treated as topics. The algorithm is designed to handle large datasets and can be utilized to extract latent themes from textual data [45]. To evaluate the clustering results, the stability of the clusters is measured using Equation (3).

(3) $$stability=\sum_{p\in Cluster}\left(\lambda_{p}-\lambda_{birth}\right)$$

**Figure 3:**
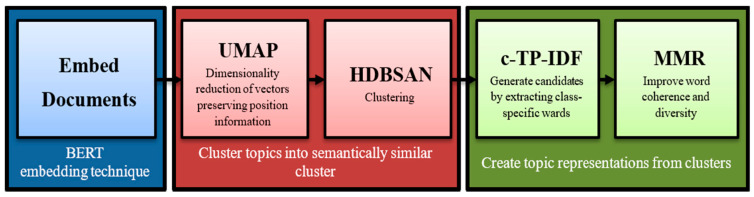
The BERTopic algorithm workflow.

The stability measures how well the clusters hold together when individual data points are removed. Specifically, *λp* represents the reciprocal of the edge weight when the root point *p* in the clustering result is separated from its cluster. *λ_birth_* represents the reciprocal of the edge weight when a new cluster is split from an existing one. BERTopic is similar to Top2Vec in that it provides continuous rather than discrete topic modeling. Therefore, the randomness of the model can produce different results when modeling repeatedly. Once the model is computed, researchers can extract the most important topics, search for keywords, and get the most relevant topics based on similarity scores. Individual topics can also be analyzed based on keywords. Finally, to facilitate analysis of a potentially large number of topics, BERTopic provides an interactive inter-topic distance map to explore individual topics [44]. Figure 3 shows the BERTopic algorithm workflow.

### 2.4. Long Short Term Memory Network

**Figure 4:**
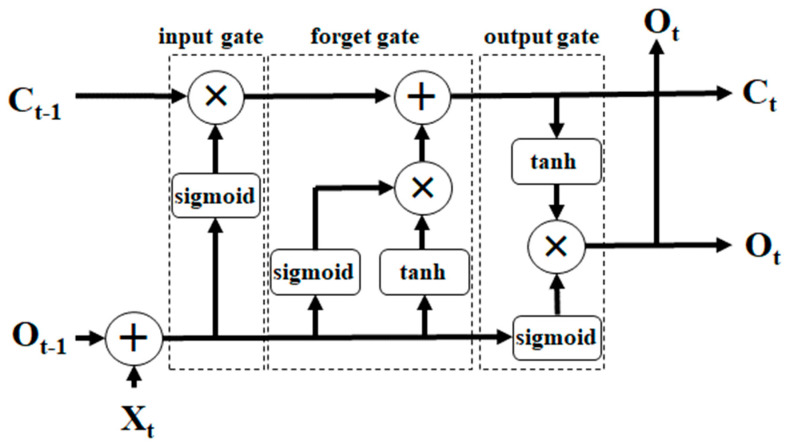
A LSTM memory cell.

The LSTM network is a powerful type of RNN that excels in capturing long-term dependencies in data. First proposed by Hochreiter and Schmidhuber in 1997, LSTM has found widespread applications in various domains [46]. Unlike conventional RNN neurons that rely solely on the previous hidden state and current input to generate new hidden states, LSTM neurons integrate cell states from multiple past time steps to update their own cell states. As memory cells, LSTM neurons can store their own cell states along with other relevant information. A typical LSTM storage unit, as shown in Figure 4, consists of three key components or gates: (1) the Forget Gate, which decides whether to replace the current cell state with the latest data, based on its output value; (2) the Input Gate, which learns how to store or update data in the appropriate cell state, taking into account various inputs, including the previous time step output (O_t−1_), current input (X_t_), and the previous time step cell state (C_t−1_); and (3) the Output Gate, which plays a crucial role in determining the data to pass to the next node in the network by considering the input and cell state, thereby facilitating the flow of information through the LSTM network. In Figure 4, the “+” and “×” symbols represent key operations within the LSTM unit. The addition operation updates the cell state by combining information from the forget gate and input gate. The element-wise multiplication is employed by the forget gate to determine how much of the previous cell state should be retained, and by the output gate to control how much of the cell state is passed to the next hidden state. These operations are essential for regulating the flow of information through the network and ensuring the effective updating and propagation of cell states across time steps.

LSTM networks, with their ability to capture dependencies in time-series data, provide a robust framework for analyzing temporal features, such as those in crowdfunding success. Their dynamic adaptability to varying temporal trends stems from their capacity to learn both long- and short-term dependencies through recurrent components. The inclusion of a forget gate in LSTM networks effectively mitigates the vanishing gradient problem during backpropagation by regulating cell state weights, eliminating the need for exponential data growth or decay [47]. This feature allows LSTM networks to achieve optimal results without requiring repeated calculations, setting them apart from other types of RNNs. Furthermore, LSTM networks are less sensitive to irregularities or time lags between data points, making them particularly well-suited for statistical applications.

To enhance the performance of our LSTM model, we implemented several optimization strategies, including hyperparameter tuning and regularization techniques [48]. Specifically, we optimized key hyperparameters such as the number of hidden layers, the number of units, the learning rate, and the batch size through Grid Search and Random Search [49,50]. These optimizations enabled the LSTM to more effectively capture long- and short-term dependencies in time-series data, significantly improving the model’s predictive capabilities. Additionally, to increase the robustness of the LSTM network and reduce overfitting, we introduced Dropout as a regularization technique within the LSTM layers. Dropout, a common method in deep learning, randomly deactivates a fraction of neurons during training, which reduces the model’s dependency on specific neurons and encourages the network to learn more generalized patterns. This approach improves the model’s performance on unseen data [51]. By applying Dropout to the LSTM layers, we ensured that the model maintained a balance between learning capacity and generalization, which is crucial for accurate time-series analysis.

### 2.5. Machine Learning

Our dataset was organized and divided into two parts for training and testing the model, with 80% and 20% of the data, respectively. We developed a hospital readmission prediction model using LSTM networks and six commonly used machine learning algorithms. The selected machine learning algorithms include SVC, RF, GB, XGBoost, LightGBM, and CatBoost. All data mining tasks were performed using the Python programming language.

SVC is a robust supervised machine learning algorithm that can analyze both linear and nonlinear data for classification and regression tasks. It produces accurate predictions for binary or multiclass classifications and is resistant to data bias and variance [52]. RF is an ensemble supervised machine learning algorithm that uses decision trees as the basic classifier. It is well suited to handle datasets with missing values and can sort features by importance [53]. RF provides higher accuracy compared to a single decision tree and can also be used for variable selection. GB enhances the accuracy of various classification prediction models by training a model with poor prediction accuracy and combining the trained results with the existing model in a cumulative form. This study employed the scikit-learn library to achieve gradient boosting [54]. XGBoost is a scalable end-to-end tree boosting system that can handle missing data efficiently and can build an assembly of weak prediction models into an accurate one. It generates a series of decision trees during training and can deal with missing values by including a default orientation for missing values in each tree node [55]. LightGBM is a powerful gradient boosting decision tree learning algorithm that has demonstrated exceptional performance in feature selection, classification, and regression analysis. It uses histogram algorithms and leaf-by-leaf strategies with depth constraints to improve prediction accuracy while minimizing memory usage and accelerating predictions [56]. CatBoost is an improved version of the gradient boosting decision tree algorithm that can handle categorical features well. It processes categorical features at training time rather than preprocessing time and uses a more efficient mode to compute leaf values during tree structure selection, reducing overfitting. CatBoost is a GBDT-based machine learning algorithm that can be used for different tasks such as binary or multiclass classification and regression [57].

### 2.6. Performance Evaluation

To make a thorough comparison of the impact of the integration of quantitative and text data on the prediction of mortality in ICU patients, in this study, five different metrics were chosen as evaluation tools for modeling. These included AUROC, Precision, Recall, F1-Score, and Accuracy.

(4) $$Precision=PPV=\frac{TP}{TP+FP}$$

(5) $$Recall=TPR=\frac{TP}{TP+FN}$$

(6) $$F1-score=\frac{2*Precision*Recall\;}{Precision+Recall\;}$$

(7) $$Accuracy=\frac{TP+TN}{TP+FP+TN+FN}$$

In predictive modeling, performance evaluation metrics play a crucial role in assessing the accuracy and effectiveness of the model. Four common metrics used in binary classification tasks are true positives (TP), true negatives (TN), false positives (FP), and false negatives (FN). Precision refers to the proportion of correct predictions in positive samples, or the proportion of positive samples among all positive samples classified. Recall, on the other hand, is the proportion of predicted positive samples among all factual positive samples. These metrics are commonly employed in identification and prediction algorithms. The F1-score, which takes into account both precision and recall, is a comprehensive measure of model performance. It is often used to evaluate the precision of judgement algorithms. Another important performance metric is accuracy, which measures the proportion of correct predictions made by the model over all samples. It is calculated as the ratio of correctly classified samples to the total number of samples in the test dataset. In the field of diagnostic testing, the area under the receiver operating characteristic (AUROC) curve is the most commonly used metric for evaluating the performance of a diagnostic tool. The AUROC curve is a graphical representation of the relationship between the true positive rate (TPR) and the false positive rate (FPR) across all possible classification thresholds. It provides a comprehensive measure of classifier performance and assigns equal importance to all instances, regardless of the positive label nature. The ROC curve is constructed using FPR on the *x*-axis and TPR on the *y*-axis.

## 3. Results

The study utilized the MIMIC-III database for data preprocessing and finally used the ICU admission records of 36,232 patients, out of which 3836 ICU patients were readmitted within 30 days of their discharge, indicating a readmission rate of 10.59%. Table 1 presents patient demographic information, revealing an average patient age of 64 years, with 56.7% of patients being male. Furthermore, medical emergencies accounted for over 81% of hospitalizations, with 37.5% of patients being admitted to the medical ICU. Medicare was the primary insurance provider for over 55% of patients. More than half of the patients opted for home recuperation after discharge. Table 1 also provides the mean values of the study variables.

### 3.1. Prediction of the Readmission

The k-fold cross-validation method is commonly employed to evaluate the performance of trained models. In this approach, the dataset is divided into k equally sized parts, and the performance is measured by averaging the test results across all iterations. This method allows the model to be trained and validated on all instances of the entire dataset, providing more accurate predictions with reduced bias. However, it can be computationally intensive and time-consuming. In our study, we predicted the 30-day readmission rate based on data collected during the last 24 h before patient discharge, along with the discharge summaries prepared by healthcare providers for ICU admissions. We utilized a 10-fold cross-validation strategy, a well-established approach for assessing model performance. The dataset was divided into 10 equal subsets, with the model being iteratively trained on nine subsets while the remaining subset was used for validation. This process was repeated 10 times, with each subset serving as the validation set once, allowing us to calculate the average performance across all iterations. This technique effectively reduces bias and variance, providing more reliable predictions and mitigating the risk of overfitting by ensuring that the model is evaluated across multiple data subsets. Additionally, by allocating 80% of the data for training and 20% for testing and adhering to strict validation and regularization protocols, we ensured that our model not only performed optimally on the training data but also maintained its predictive accuracy when applied to unseen data. Table 2 lists the AUROC obtained for the LSTM and the six different machine learning methods employed in our study.

To investigate the effect of adding BERTopic topics on the prediction of readmission for ICU patients within thirty days of discharge, we compared the performance of models using only quantitative data with those using both quantitative and BERTopic data within 24 h of ICU admission. Our results show that adding patients’ text data discharge summary can improve the accuracy of readmission rate prediction. For a detailed overview of the model performance using different classifiers, please refer to Table 2: AUROC and Confidence Intervals for Different Classifiers.

**Figure 5:**
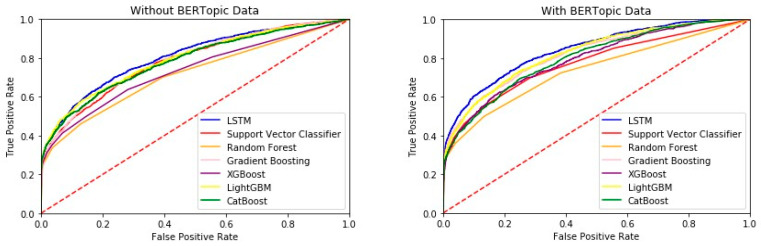
ROC curves for the different classifiers.

Figure 5 shows that LSTM with the BERTopic data can further improve prediction accuracy. Our findings demonstrate that a model developed by integrating quantitative and text data from ICU patients can accurately predict post-discharge readmission rates. Furthermore, our models using both quantitative and text data generally outperform models using only quantitative data. BERTopic-LSTM is the best models for predicting ICU patient readmission rates. The AUROC is a single measure used to summarize the overall performance of the model, with a value of 1.0 representing a perfect classifier and 0.5 denoting a model that performs no better than random guessing. In Figure 5, the red dashed line represents the performance of a random classifier, serving as the baseline with an AUROC of 0.5. In our study, the ROC curves for each classifier, including those enhanced with BERTopic data, illustrate the improvement in discriminative ability brought about by integrating textual data from patient discharge summaries. This visual representation helps clarify how well the model can distinguish between patients who will be readmitted within 30 days and those who will not, thereby supporting more effective clinical decision-making, as shown by the data in Figure 5. The model achieves an AUROC of 0.7994. Our study highlights the value of basic patient observation and judgment during hospitalization, which significantly affects the accuracy of model predictions. By taking text data as input, higher-level concepts not present in physiological data can be accessed. Overall, our results suggest that integrating quantitative and text data from hospital ICU patients can accurately predict post-discharge readmission rates.

Table 3 summarizes the performance of the LSTM and the six different machine learning models in predicting the readmission rate of ICU patients within 30 days after discharge, using four different evaluation metrics: Precision, Recall, F1-Score, and Accuracy. The results indicate that the addition of the BERTopic method to process text data improves the predictive accuracy of the models compared to using only quantitative data. The BERTopic-LSTM model achieved the highest accuracy performance (0.9316), followed by Random Forest (0.9290), CatBoost (0.8961), and XGBoost (0.8868).

### 3.2. Text Data Analysis

**Figure 6:**
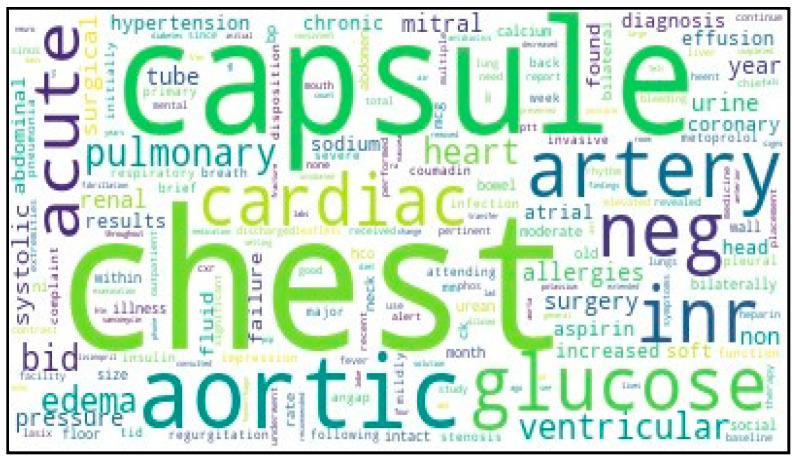
Word cloud of discharge summary from the MIMIC-III database.

In the MIMIC-III database, each hospitalization of ICU patients has multiple text records recorded in the NOTEEVENTS table. To predict whether patients will be readmitted within 30 days after discharge, we use the discharge summary as the main text data source. Based on the discharge summaries from the MIMIC-III database, we created a word cloud to summarize the main features of patients, aiming to provide further clinical insights and assist in the management of their conditions. Figure 6 displays the resulting word cloud.

The BERTopic method is employed to generate basic “topics” that serve as input variables to build prediction models. To determine the optimal number of topics and the final model to be used, we follow Baird et al. and Abuzayed et al.’s method [58,59]. The analysis results show that 10 topics provide the best prediction performance. Table 4 provides a comprehensive depiction of all 10 topics and their corresponding keywords as processed through our database.

### 3.3. Feature Importance

**Figure 7:**
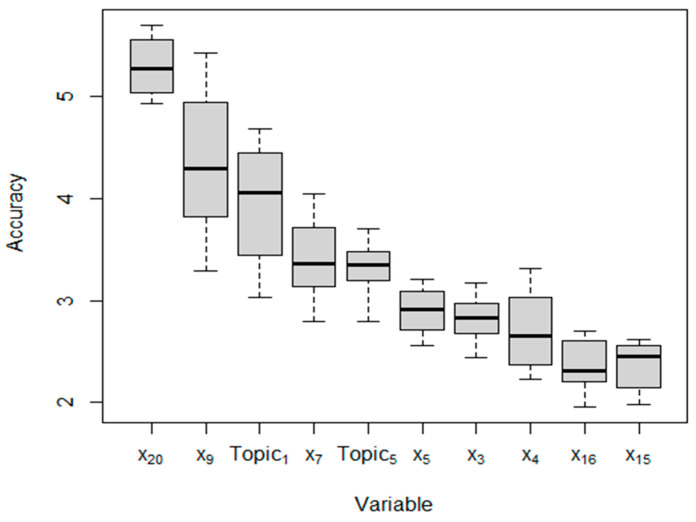
The variable importance obtained by the BERTopic-LSTM model.

In the study of predicting the readmission of ICU patients 30 days after discharge, the predictive variables may include the patient’s basic information, medical records, physiological indicators, test results, imaging data, and many other variables, and only some of them may be important for predicting readmission 30 days after discharge. To gain insights into the predictions made by deep learning models, it is crucial to understand the significance of different features within the models. However, interpreting deep learning models can be challenging due to their complex structures and numerous parameters. To assess the importance of features in LSTM models, we utilized the permutation importance method, originally proposed by Breiman [53]. This method is applicable for both traditional machine learning models and deep learning methods. In our study, we employed the Python package Eli5 [60] to implement the permutation importance method. In permutation importance, each feature’s columns are shuffled one at a time. After shuffling, the model is re-evaluated with the data of one incorrectly shuffled feature. If the model’s performance (measured by accuracy) significantly decreases after shuffling a particular feature, it indicates that the shuffled feature has high predictive power. On the other hand, if the model’s performance remains unaffected, the shuffled feature is assumed to have little or no predictive power. This process is repeated for all features in the feature space. To account for possible variability due to randomization, we calculated the permutation importance scores ten times and averaged them in our study. The obtained average variable importance for the best model in each cluster is presented in Figure 7, with the *y*-axis indicating the increase in accuracy.

Based on Figure 7, the permutation importance analysis revealed that the increases in values of accuracy were relatively small. However, instead of focusing solely on the raw average increase values, we examined the average importance ranking of each feature. From Figure 7, it was observed that the top five most important variables were X_20_ (Discharge Location), X_9_ (Non-Invasive Blood Pressure), Topic_1_ (Heart Surgery), X_7_ (Blood Urea Nitrogen), and Topic_5_ (Kidney Disease). Among these variables, X_20_ (Discharge Location) and Topic_1_ (Heart Surgery) were found to have the most significant influence on predicting the patient readmission. In contrast, X_7_ (Blood Urea Nitrogen) and Topic_5_ (Kidney Disease) had a comparatively lower impact on the prediction of the patient readmission. It is worth mentioning that the top five most important variables included two topic variables, indicating that the topic variables generated by the BERTopic method had a substantial impact on the patient readmission prediction.

Overall, the results of this study demonstrate the potential of combining machine learning tools and BERTopic-LSTM technology to identify a comprehensive set of critical predictors for ICU patient readmission. By utilizing both quantitative and text data sources, healthcare providers can gain a deeper understanding of the factors that contribute to readmission and develop targeted interventions to improve patient outcomes. Specifically, in practical applications, the use of machine learning to screen important features can help doctors or researchers more comprehensively understand the operating principles of predictive models and improve trust in predictive results. In addition, screening out important features can reduce the dimensionality of data, thereby reducing model complexity, reducing computing time and resource consumption, improving prediction efficiency and stability, and making it more suitable for practical applications.

## 4. Discussion

### 4.1. Principal Findings

This study makes several significant contributions. First, it applies BERTopic technology to analyze textual data in EHRs and generate relevant thematic variables [61,62]. Second, it integrates quantitative and textual clinical records, leveraging an LSTM network and six machine learning models to predict patient readmission within 30 days of discharge. Recent related research provides insight into how our findings impact current ICU treatment strategies and patient clinical prognosis [63,64,65]. Third, the importance of the generated variables can be further analyzed through machine learning methods to evaluate key factors for patient readmission, thereby taking potential preventive measures in medical practice [66,67,68]. These improvements and the integration of the latest research underscore the relevance of our research to the ongoing development of the field. These results can improve healthcare professionals’ predictions of patient readmissions and provide patients, patients’ families, and healthcare professionals with more information for clinical decision-making.

#### 4.1.1. BERTopic-LSTM Model

We utilized the BERTopic method to generate ten distinct topics, each with its corresponding set of keywords. To ensure their relevance to the medical domain, we corroborated these topics with existing literature. To further evaluate the clinical validity of our findings, we selected at least five high-probability keywords from each of the ten topics and examined their co-occurrence in the medical literature. Our investigation revealed that the majority of these condition pairs are well-established, which supports our topic-keyword with readmission associations.

Using BERTopic, we found that readmissions and topics are corroborated in many academic studies. For instance, Topic_1_ pertains to heart surgery and includes keywords such as chest, artery, aortic, cardiac, ventricular, coronary, and mitral [69]. Topic_2_ is related to drug abuse and infection, featuring terms like urine, alcohol, allergies, overdose [70]. Topic_3_ concerns liver disease and comprises keywords such as liver, bleeding, abdominal, bowel, cirrhosis, and bleed [71]. Topic_4_ relates to respiratory conditions and features keywords such as respiratory, pulmonary, bronchoscopy, chest, lung, pneumonia, and breath [72]. Topic_5_ focuses on kidney disease and contains terms like renal, dialysis, urine, kidney, catheter, and hemodialysis [73]. TOPIC_6_ is about Biliary Tract Disorders, and keywords include biliary, bile, abdominal, gallbladder, cholangitis [74]. Topic_7_ pertains to diabetes management, with keywords including insulin, glucose, diabetes, diabetic, humalog, lantus, and hyperglycemia [75]. Lastly, Topic_9_ concerns cancer treatment and features keywords such as lymphoma, melanoma, metastatic, mass, cell, and chemotherapy [76]. Overall, our study demonstrates the usefulness of the BERTopic method in identifying relevant topics and associated keywords in the medical domain. These findings have significant implications for healthcare practitioners and researchers as they provide a framework for exploring and understanding complex medical conditions.

In comparison, BERTopic offers several advantages. First, it is based on the BERT model, a deep neural network model that can learn advanced semantic features from large amounts of data, enabling it to better distinguish topics in text data. Additionally, BERTopic is particularly effective at handling long text data because the BERT model is designed to consider the context of words, allowing it to better understand their meanings [59]. Another benefit of BERTopic is that it can automatically adjust parameters without requiring manual adjustment, making it more convenient to use [27]. Abeer and Hend experimented with BERTopic using different pretrained Arabic language models as embeddings on 108,789 Arabic documents. The results of topic modeling techniques are evaluated using the Normalized Pointwise Mutual Information (NPMI) metric. In the context of medical big data applications, BERTopic’s ability to accurately distinguish themes in patients’ medical records can provide more precise and personalized healthcare recommendations.

The superior performance of the LSTM model in this study is attributed to its specialized architecture and the strategic application of optimization techniques. Unlike traditional recurrent neural networks, LSTM’s memory cells and gating mechanisms enable it to effectively capture both short- and long-term temporal dependencies [77]. Through targeted hyperparameter tuning, including adjustments to the number of layers, units, learning rate, and batch size, the model’s ability to learn dataset-specific temporal patterns was significantly enhanced, leading to improved predictive accuracy [78]. Additionally, the use of Dropout as a regularization technique mitigated overfitting by reducing reliance on specific neurons, thus promoting better generalization to unseen data. The combination of LSTM’s architectural strengths, optimized hyperparameters, and Dropout regularization demonstrated the model’s robustness in time-series forecasting. The integration of BERTopic for textual data analysis further improved the model’s predictive capability. This synthesis of structural advantages and rigorous optimization explains the BERTopic-LSTM model’s superior performance in predicting ICU patient readmission rates.

#### 4.1.2. Readmission

In recent years, the avoidable readmission rate has become a critical measure of the quality of care provided by hospitals. To understand readmission rates, many studies have focused on building predictive models using healthcare data, including patient demographics, social characteristics, hospital utilization, medications, procedures, pre-existing conditions, laboratory tests, and insurance claims records. However, existing machine learning-based models have reported consistent but mediocre performance, with AUC scores ranging from 0.60–0.70. Deng et al. applied three time series deep learning models to predict the ROC curve for predicting the 30-day readmission rate. The readmission prediction AUC of the three deep learning models of RNN, GRU, and LSTM reached 0.625, 0.631, and 0.635 [79]. Xue et al., using grouped physiological and drug trends, predicted the best AUROC of ICU patients being readmitted within 30 days after discharge as 0.66 [80]. Alvaro et al. also predicted the readmission rate of patients in the MIMIC-III database, achieving a best accuracy of 86.4% and AUROC of 0.64 [81]. Wang and Cui proposed to use CNN to automatically learn features, and the AUC of the proposed model was 0.70 [82]. Min and Yu demonstrated that state-of-the-art deep learning models fail to improve prediction accuracy, with a best AUC of 0.65 [83]. Huang and Altosaar developed a deep learning model to process clinical records and predict the associated readmission risk score (AUC of RNN = 0.694) [45].

In comparison to these traditional and deep learning models, our approach significantly enhances the accuracy of readmission rate predictions by applying the BERTopic method to analyze patient discharge summaries and integrating both quantitative and textual data from ICU patients. Our BERTopic-LSTM model achieved an AUROC score of 0.7994 within the LSTM networks, which is notably superior to the results reported in previous studies. This indicates that combining natural language processing methods with quantitative data allows for more accurate prediction of discharge readmission rates. Moreover, direct comparisons with other models demonstrate that BERTopic-LSTM excels not only in prediction accuracy but also in handling the complexity and diversity of medical textual data. By integrating BERTopic’s topic modeling with LSTM’s time-series forecasting capabilities, our study presents an innovative and effective method for readmission rate prediction, further showcasing the immense potential of natural language processing and deep learning in the context of medical big data.

#### 4.1.3. Predicting Variables

In our study, we conducted a literature review to identify the risk factors associated with ICU readmissions and incorporated these variables into our machine learning models [84,85]. Consistent with previous research, we found that physiological variables such as blood urea nitrogen, age, and length of ICU stay were significant predictors of readmission, compared to general hospital readmissions [36,79]. Additionally, other studies have highlighted the importance of medical insurance and discharge destination in predicting readmissions [86]. Our predicting models were able to identify key predictors of readmission within 30 days of ICU discharge. Using quantitative data, we found that discharge site, systolic blood pressure, temperature, blood urea nitrogen, noninvasive blood pressure, age, length of ICU stay, and length of hospital stay were important variables for predicting patient readmission. Furthermore, we used BERTopic technology to process text data, which allowed us to identify additional variables that were significant in predicting readmissions. These variables were categorized into topics, including cardiac surgery, substance abuse, infection, diabetes management, and hand surgery.

Our study provides valuable insights into the factors that influence ICU readmissions and highlights the potential of machine learning and natural language processing techniques for analyzing complex healthcare data. By identifying key predictors of readmissions, our findings could inform clinical decision-making and help healthcare providers reduce the risk of readmissions, ultimately improving patient outcomes.

### 4.2. Limitations

Recognizing the limitations of current research is critical. Our study is based on patient data from the single-institution MIMIC-III. Since this study was limited to ICU care data collected in large institutions in urban areas, the findings may not be applicable to ICU patients in smaller institutions. More comprehensive results and validation can be obtained by comparing these findings with data collected from rural or other small healthcare settings. Additionally, the model’s performance in other critical care settings may suffer due to the lack of high-quality care records for large numbers of patients. Information recorded by physicians during patient consultations is valuable for disease and treatment research. However, spelling mistakes and other inaccuracies common in these medical notes can affect the quality of interpretation, resulting in less accurate predictions [87]. Another significant limitation is the absence of proper external validation, which affects the overall credibility and generalizability of our predictive model. Furthermore, our study only included clinical data from the 24 h prior to ICU discharge and the discharge summaries written by doctors. This limited scope prevented us from assessing changes during the entire ICU stay, including more comprehensive clinical records and test results, which could have provided a more complete picture and potentially influenced the predictive outcomes.

Finally, the challenge of imbalanced datasets poses significant limitations. In medical data such as mortality and readmission rates, class distributions are often highly skewed, resulting in an imbalanced dataset. This imbalance can negatively impact the performance of traditional learning algorithms, reducing their prediction accuracy. To solve this problem, future research should explore and implement advanced techniques for handling imbalanced data. In addition to the SMOTE technology used in this study, other methods such as ADASYN (Adaptive Synthetic Sampling) [88] and various resampling methods can be tried. These techniques hold considerable promise, especially in the context of clinical diagnostic prediction using actual patient data. Through further research and rigorous testing, we aim to enhance our academic and practical contributions to the field of healthcare data analytics.

## 5. Conclusions

Predicting the likelihood of readmission among patients in intensive care units (ICUs) holds paramount importance in curbing healthcare expenditures and enhancing patient outcomes. By identifying individuals with a heightened risk of readmission, healthcare providers can devise bespoke treatment strategies and proactive interventions, such as tailored care regimens and vigilant health surveillance. This approach not only alleviates the strain on healthcare infrastructures but also ensures the prompt and appropriate delivery of care to patients. Through the employment of predictive models targeting high-risk cohorts, including the elderly, immunocompromised individuals, and those afflicted with chronic ailments, medical practitioners can effectively mitigate the specter of readmission, thereby augmenting both the efficiency and efficacy of healthcare services.

In our research, we leveraged unstructured data from the MIMIC-III NOTEEVENTS table, with a focus on discharge summaries that encapsulate comprehensive patient information recorded during ICU stays. These summaries are pivotal as they integrate detailed accounts of the patient’s medical history, treatment during the stay, and the conditions at discharge, offering a holistic view of each patient’s health status. The utilization of discharge summaries is critical for our study as it allows for a nuanced analysis of factors contributing to the likelihood of readmission within 30 days post-discharge. By analyzing these summaries, our model gains the ability to extract key predictive indicators, significantly enhancing the accuracy of readmission risk assessments.

We combine quantitative and textual data collected during patients’ ICU stay. Within the purview of this paper, we introduce an innovative methodology for prognosticating ICU patient readmission from EHRs utilizing a hybrid approach integrating BERTopic-LSTM networks. By amalgamating the merits of unsupervised topic modeling and supervised deep learning, our model adeptly captures the intricate interplay between patient attributes and readmission risk. Our methodology achieves a multitude of critical objectives and advantages for both healthcare practitioners and patients alike. Firstly, we unearth potential topics latent within patient medical records, thereby transmuting copious amounts of textual data into cogent and interpretable themes. This facilitates physicians in attaining a deeper comprehension of a patient’s clinical trajectory and treatment continuum. Secondly, the personalization of patient prognostication is rendered feasible through the conversion of information encapsulated in the patient discharge summaries into a topic vector. By prognosticating patient readmission predicated on distinct topics, clinicians can formulate more tailored treatment strategies and exercise enhanced patient management. Thirdly, our approach ameliorates the standard of medical care by prognosticating patient readmission and enabling early intervention to curtail the readmission rate. Lastly, our methodology curtails the financial ramifications associated with readmission by facilitating early intervention and patient management.

Practically, the application of BERTopic technology to topic modeling and analysis of patient discharge summaries furnishes healthcare professionals with an enhanced comprehension of patient conditions, facilitates the formulation of personalized treatment regimens, and engenders more efficient resource allocation. Moreover, our predictive model, which integrates both quantitative and textual data from patient records, provides healthcare personnel with a powerful tool to identify patients at high risk of readmission within 30 days post-discharge. This model enables healthcare providers to implement targeted interventions, optimize follow-up care, and make data-driven decisions that improve patient outcomes. By incorporating these insights into routine clinical practice, medical institutions can better manage patient care, dispense superior services, and ultimately enhance the quality of medical care while simultaneously mitigating costs.

## Figures and Tables

**Table 1:** Selected patient demographic information.

	Readmission Within 30 Days	No Readmission Within 30 Days	Total
Patient	3836 (10.59%)	32,396 (89.41%)	36,232 (100%)
Age	70.21 [60.22–82.87]	63.28 [52.40–76.68]	64.01 [53.12–77.49]
Gender (Male)	2146 (55.94%)	18,395 (56.78%)	20,541(56.69%)
Admission Type			
Emergency	3533 (92.10%)	26,058 (80.43%)	29,591 (81.67%)
Elective	236 (6.15%)	5531 (17.07%)	5767 (15.92%)
Urgent	67 (1.75%)	807 (2.49%)	874 (2.41%)
Last Care Unit			
Coronary Care Unit	531 (13.84%)	4302 (13.28%)	4833 (13.34%)
Cardiac Surgery Recovery Unit	368 (9.59%)	7364 (22.73%)	7732 (21.34%)
Medical ICU	2043 (53.26%)	11,540 (35.62%)	13,583 (37.49%)
Surgical ICU	579 (15.09%)	5522 (17.04%)	6101 (16.84%)
Trauma Surgical ICU	315 (8.21%)	3668 (11.32	3983 (10.99%)
Insurance Type			
Self Pay	18 (0.47%)	306 (0.94%)	324 (0.89%)
Government	46 (1.20%)	959 (2.96%)	1005 (2.77%)
Medicare	2749 (71.66%)	17,288 (53.36%)	20,037 (55.30%)
Medicaid	268 (6.99%)	2860 (8.83%)	3128 (8.63%)
Private	755 (19.68%)	10,983 (33.90%)	11,738 (32.40%)
Discharge Location			
Home	999 (26.04%)	18,496 (57.09%)	19,495 (53.81%)
Rehab/Distinct Part Hospital	1008 (26.28%)	8019 (24.75%)	9027 (24.91%)
Skilled Nursing Facility	811 (21.14%)	5742 (17.72%)	6553 (18.09%)
Hospice	244 (6.36%)	131 (0.40%)	375 (1.04%)
Dead/Expired	774 (20.18%)	8 (0.02%)	782 (2.16%)
Variable Value			
Heart Rate	84.75 ± 14.72	82.78 ± 13.4	82.99 ± 13.56
Respiratory Rate	20.21 ± 4.4	19.23 ± 3.84	19.33 ± 3.92
Diastolic Blood Pressure	60.46 ± 42.7	62.59 ± 53.86	62.36 ± 52.79
Systolic Blood Pressure	120.87 ± 19.34	123.21 ± 48.76	122.96 ± 46.54
Temperature	97.91 ± 3.01	98.11 ± 3.95	98.09 ± 3.86
Oxygen Saturation	96.53 ± 4.46	96.53 ± 4.69	96.53 ± 4.66
Blood Urea Nitrogen	32.87 ± 24.83	22.48 ± 17.6	23.58 ± 18.77
Creatinine	1.57 ± 1.59	1.26 ± 1.92	1.3 ± 1.89
Non-invasive Blood Pressure	76.78 ± 11.54	79.16 ± 9.68	78.91 ± 9.92
Glucose	122.95 ± 63.46	119.45 ± 57.61	119.82 ± 58.26
White Blood Cell	10.82 ± 9.14	10.1 ± 4.98	10.19 ± 5.57
GCS Eye	3.69 ± 0.63	3.87 ± 0.32	3.85 ± 0.37
GCS Motor	5.69 ± 0.83	5.91 ± 0.37	5.89 ± 0.45
GCS Verbal	3.99 ± 1.44	4.55 ± 0.94	4.49 ± 1.02
ICU Length of Stay	5.90 [1.85–6.05]	4.14 [1.57–4.18]	4.33 [1.60–4.33]
Hospital Length of Stay	12.50 [5.35–15.45]	10.62 [4.86–12.62]	10.82 [4.89–12.88]

**Table 2:** AUROC and confidence intervals for different classifiers.

Method	Without BERTopic Data	With BERTopic Data
	AUROC (95% CI)	AUROC (95% CI)
LSTM	0.7532 (0.7490–0.7574)	0.7994 (0.7957–0.8031)
Support Vector Classifier	0.6987 (0.6945–0.7029)	0.7092 (0.7056–0.7128)
Random Forest	0.6614 (0.6536–0.6692)	0.6807 (0.6743–0.6871)
Gradient Boosting	0.7193 (0.7151–0.7235)	0.7441 (0.7397–0.7485)
XGBoost	0.6921 (0.6899–0.6943)	0.7265 (0.7232–0.7298)
LightGBM	0.7205 (0.7185–0.7225)	0.7463 (0.7397–0.7529)
CatBoost	0.7065 (0.7003–0.7127)	0.7322 (0.7229–0.7415)

AUROC, area under the receiver operating characteristic curve; CI, confidence interval.

**Table 3:** Diagnostic precision, recall, F1-score, and accuracy.

Dataset	Method	Precision	Recall	F1-Score	Accuracy
Without BERTopic Data	LSTM	0.8802 ± 0.0082	0.7834 ± 0.0069	0.8558 ± 0.0065	0.8857 ± 0.0027
	Support Vector Classifier	0.2485 ± 0.0032	0.6221 ± 0.0049	0.3552 ± 0.0041	0.7579 ± 0.0027
	Random Forest	0.8656 ± 0.0149	0.3126 ± 0.0124	0.4592 ± 0.0154	0.9171 ± 0.0012
	Gradient Boosting	0.2778 ± 0.0046	0.6387 ± 0.0053	0.3872 ± 0.0047	0.7826 ± 0.0035
	XGBoost	0.4204 ± 0.0100	0.4609 ± 0.0042	0.4396 ± 0.0048	0.8736 ± 0.0022
	LightGBM	0.3632 ± 0.0078	0.5568 ± 0.0017	0.4395 ± 0.0052	0.8473 ± 0.0033
	CatBoost	0.4468 ± 0.0087	0.4855 ± 0.0116	0.4652 ± 0.0038	0.8795 ± 0.0015
With BERTopic Data	LSTM	0.9355 ± 0.0072	0.8286 ± 0.0088	0.8788 ± 0.0063	0.9316 ± 0.0024
	Support Vector Classifier	0.2497 ± 0.0063	0.6391 ± 0.0045	0.3582 ± 0.0073	0.7635 ± 0.0017
	Random Forest	0.8610 ± 0.0109	0.3072 ± 0.0082	0.4528 ± 0.0095	0.9290 ± 0.0024
	Gradient Boosting	0.2918 ± 0.0045	0.6829 ± 0.0115	0.4088 ± 0.0038	0.7934 ± 0.0045
	XGBoost	0.4644 ± 0.0028	0.5007 ± 0.0048	0.4819 ± 0.0009	0.8868 ± 0.0003
	LightGBM	0.3852 ± 0.0061	0.6014 ± 0.0101	0.4696 ± 0.0065	0.8572 ± 0.0022
	CatBoost	0.5057 ± 0.0117	0.5247 ± 0.0132	0.5150 ± 0.0107	0.8961 ± 0.0025

**Table 4:** Selected variable topics and keywords for dataset.

No	Topic	Keywords
1	Heart Surgery	chest, artery, aortic, cardiac, ventricular, coronary, mitral, surgery, heart, intact, allergies, stenosis, surgical, systolic, atrial
2	Substance Abuse and Infection	urine, alcohol, allergies, acute, capsule, infection, edema, overdose, disposition, withdrawal, fever, diagnosis
3	Liver Disease	liver, bleeding, abdominal, bowel, cirrhosis, capsule, egd, bleed, tube, glucose, fluid, chest, portal, hepatic, varices, abdomen, ascites, acute
4	Respiratory Conditions	respiratory, pulmonary, bronchoscopy, chest, lung, pneumonia, breath, copd, capsule, tube, cough, inhalation, prednisone, albuterol, cxr, lobe, failure, chronic, tracheal
5	Kidney Disease	renal, dialysis, urine, kidney, catheter, capsule, hemodialysis, failure, fluid, acute, glucose, esrd, infection, calcium, chest, chronic, insulin
6	Biliary Tract Disorders	biliary, pancreatitis, bile, abdominal, stent, fluid, gallbladder, cholangitis, pancreatic, acute, liver, cbd, ast, alt, lipase, tube, abdomen, drain, capsule, glucose
7	Diabetes Management	insulin, glucose, diabetes, diabetic, humalog, lantus, sugars, anion, nausea, subcutaneous, capsule, acute, calcium, renal, unitml, hyperglycemia, sugar, hco, regimen
8	Neurological Disorders	seizure, seizures, eeg, dilantin, keppra, head, neurology, ativan, intubated, urine, bilaterally, generalized, mental, capsule, alcohol, glucose, impression, further
9	Cancer Treatment	lymphoma, melanoma, metastatic, mass, renal, cell, chemotherapy, chest, edema, capsule, brain, pulmonary, treatment, cancer, mri, acute, biopsy, received, calcium
10	Hand Surgery	finger, hand, amputation, repair, ring, radial, injury, wrist, long, plastic, capsule, distal, surgery, postoperatively, signs, proximal, clinic, laceration, dilaudid, vital

## Data Availability

The original contributions presented in the study are included in the article; further inquiries can be directed to the corresponding author.

## Appendix A. Selected Variables

	**Feature**	**Item ID**	**Table**
X_1_	Heart Rate	211, 220045	Chartevents
X_2_	Respiratory Rate	220210, 224689, 224690, 618, 615, 614	Chartevents
X_3_	Diastolic Blood Pressure	8368, 8441, 20180, 20051	Chartevents
X_4_	Systolic Blood Pressure	51, 455, 220179, 220050	Chartevents
X_5_	Temperature	676, 223762, 678, 223761	Chartevents
X_6_	Oxygen Saturation	220277, 646	Chartevents
X_7_	Blood Urea Nitrogen	225624, 781	Chartevents
X_8_	Creatinine	220615, 791	Chartevents
X_9_	Non-Invasive Blood Pressure	220181	Chartevents
X_10_	Glucose	807, 225664, 811	Chartevents
X_11_	White Blood Cell	1127, 861, 1542, 220546	Chartevents
X_12_	GCS Eye	184, 220739	Chartevents
X_13_	GCS Motor	454, 223901	Chartevents
X_14_	GCS Verbal	723, 223900	Chartevents
X_15_	Age		Admissions, Patients
X_16_	ICU Length of Stay		Icustays
X_17_	Hospital Length of Stay		Admissions
X_18_	Gender		Patients
X_19_	Admission Type		Admissions
X_20_	Discharge Location		Admissions
X_21_	Insurance		Admissions
X_22_	Last ICU Type		Icustays
